# A Novel Combination of Postbiotics and Essential Oil Compounds Supports a Consistent Improvement in Broiler Performance

**DOI:** 10.3390/ani16020209

**Published:** 2026-01-10

**Authors:** Vivek A. Kuttappan, Gregory S. Archer, Yann Fournis, Marc Decoux

**Affiliations:** 1 Cargill Animal Nutrition, Cargill Inc., 15407 McGinty Road West, Wayzata, MN 55391, USA; yann_fournis@cargill.com (Y.F.); marc_decoux@cargill.com (M.D.); 2 Department of Poultry Science, Texas A&M University, 101 Kleberg Center, MS 2472, College Station, TX 77843, USA; gregory.archer@ag.tamu.edu

**Keywords:** phytogenics, gut health, microbiome maturation, *Saccharomyces cerevisiae*, breast meat yield

## Abstract

Recent innovations in poultry feed technology have emphasized the role of postbiotics and phytogenics as promising strategies to strengthen gut health and improve overall performance in broilers. Within this context, the current study evaluated the effectiveness of Biostrong™ Dual (Cargill Inc., Cedar Rapids, IA, USA), a novel product that integrates *Saccharomyces cerevisiae* fermentation-derived postbiotic products (SCFPs) with a proprietary blend of essential oil compounds (EOCs). The objective was to determine whether this dual formulation could consistently enhance growth, feed efficiency, and carcass quality across multiple production phases. To test this, three independent trials were conducted using commercial broiler strains. Birds were allocated to either a control group (CON) receiving a basal diet or a treatment group (DUAL) receiving the same diet supplemented with 0.4 kg/MT of Biostrong™ Dual. Each trial employed a randomized block design with 24 replicates per treatment and 16–25 birds per replicate. Results consistently demonstrated that DUAL improved (*p* < 0.05) body weight and the cumulative feed conversion ratio (cFCR) at 42 days. Pooled analysis revealed body weight gains of 5.5%, a cFCR improvement of 5 points, increased feed intake, and a 0.86% rise in breast meat yield. Additionally, one trial showed reduced footpad lesion scores. Collectively, these findings highlight Biostrong™ Dual as a valuable nutritional intervention to optimize productivity and carcass quality in poultry production and further research is needed to understand the mode of action of the product.

## Simple Summary

The present study addressed the challenge of enhancing poultry gut health, a key factor in digestion, nutrient utilization, and overall growth. Researchers evaluated Biostrong™ Dual (Cargill Inc., Cedar Rapids, IA, USA), a feed supplement composed of yeast fermentation products (postbiotics) and essential oil compounds (phytogenics), to determine its impact on broiler chicken performance. Across three large trials, birds receiving Biostrong™ Dual demonstrated faster growth, improved feed-to-body weight conversion, more consistent feed intake, and slightly higher breast meat yield compared to those on a standard diet. In one experiment, the supplement also reduced footpad lesions. Overall, the findings show that combining postbiotics and phytogenics can improve production efficiency, offering farmers a practical tool to achieve stronger performance results under varying conditions.

## 1. Introduction

The global population is projected to reach 8.8 billion by 2034 [1]. This growth intensifies the need for sustainable food systems to ensure food security. Rising affluence and urbanization are shifting diets toward protein-rich foods [1], creating pressure on animal agriculture to meet demand responsibly. Poultry, due to affordability, efficiency, and a lower environmental footprint, has become central to global protein supply [2]. Meeting future demand requires balancing environmental care, economic viability, and social responsibility [3]. Key strategies include improving efficiency, reducing environmental impact, ensuring welfare, and adopting innovation [4]. Gut health plays a vital role in these strategies, supporting digestion, nutrient absorption, and overall system function [5].

Nutritional interventions are widely used to improve gut health. Options include prebiotics, probiotics, postbiotics, phytogenics, and organic acids. Yet, results often vary across farms, raising concerns about consistency [6]. Deeper study of feed ingredient mechanisms shows that single technologies may not maximize benefits [6,7]. Combining approaches may provide more reliable outcomes.

Postbiotics and essential oil compounds are two feed ingredients with potential synergy. Postbiotics are increasingly applied in humans and animals, offering anti-inflammatory, antibacterial, immunomodulatory, antioxidant, and other health benefits [8,9]. They are defined as a “preparation of inanimate microorganisms and/or their components that confer a health benefit on the host” [10]. Postbiotic products contain proteins, peptides, oligosaccharides, vitamins, minerals, enzymes, and other bioactive compounds that support host health [11]. Unlike prebiotics and probiotics, postbiotics remain stable during feed processing and storage, ensuring bioavailability and consistent effects [12].

Among postbiotics, *Saccharomyces cerevisiae* fermentation-derived products (SCFPs) are widely studied and used [11]. SCFPs benefit poultry by enhancing stress response [13,14,15], modulating immunity [16,17,18,19,20], and shaping the gut microbiome [21,22,23,24,25,26,27,28,29]. Essential oils, meanwhile, support enzymatic activity, provide antioxidant effects, and help control harmful gut bacteria [30,31,32,33]. A preliminary study showed that combining SCFPs with essential oil compounds (Biostrong™ Dual, Cargill Inc., Cedar Rapids, IA, USA) improved growth, metabolites, and antioxidant profiles in broiler chicks [34].

The objective of this study is to evaluate the consistency of Biostrong™ Dual across different conditions and phases of broiler production. We hypothesize that combining SCFPs with essential oils will deliver superior and consistent health benefits under different conditions compared to individual use.

## 2. Materials and Methods

Three independent experiments were conducted comparing broiler birds fed with a basal diet (CON) and the treatment group (DUAL), which was fed with on-top inclusion of Biostrong™ Dual (Cargill Inc., Cedar Rapids, IA, USA) feed ingredient with the basal diet. Previously, the dose response of the combination product was evaluated using 0.2, 0.4, and 0.8 kg/MT of inclusion in finished feed [34]. Results from the study showed that 0.4 kg/MT was the lowest dose providing maximum benefit, supporting enhanced growth performance along with improved metabolites and antioxidant profiles in broiler chicks [34]. In the present study, the experiments were conducted using the same dose (0.4 kg/MT of Biostrong™ Dual) in different research settings to evaluate the consistency of benefits of the product under various challenging conditions.

### 2.1. Experimental Design, Bird Housing, and Treatments

#### 2.1.1. Experiment 1 and 2

Experiments 1 and 2 were conducted at Texas A&M University with similar experimental designs. All procedures were carried out in accordance with the guidelines established by the Texas A&M Institutional Animal Care and Use Committee (IACUC 2019-0056) and the birds were managed according to the guidelines described in the Guide for the Care and Use of Agricultural Animals in Research and Teaching. In Experiment 1, 960-day-old Cobb 500 male broiler chicks were sourced from a commercial hatchery and randomly assigned to two treatment groups (CON and DUAL). Each treatment consisted of 24 replicate pens, with 20 birds per pen, arranged in a randomized complete block design. In Experiment 2, 1200-day-old Ross 708 male broiler chicks were obtained and similarly allocated to CON and DUAL treatments, with 24 replicate pens per treatment and 25 birds per pen. The overall study design remained consistent across both experiments. All birds received a commercial coccidiosis vaccine on the day of placement. The birds were fed with the same basal diet, except the SCFP and EOC combination feed ingredient was added on top for the DUAL treatment group, which was corn-soybean-based and met or exceeded NRC 1994 recommendations [35] in three phases: starter (0–14 d), grower (14–28 d), and finisher (28 to 42 d). All birds had *ad libitum* access to feed and water throughout the period of study and were grown on re-used litter to mimic commercial management stress conditions. Birds were housed in floor pens measuring 0.91 × 1.83 m with 0.9 ft^2^/bird floor space. The temperature of the house was set to 95 °F (35 °C) for 7 days, then 88 °F (31 °C) for 8 days after the arrival of chicks to the facility. In week 2, the house temperature was maintained at 85 °F (29 °C). Beginning at week 2, the house temperature was reduced by 5 degrees each week until ambient temperature was reached. The house received 24 h of light for the first 3 days and then 20 h of light and 4 h of darkness until study completion on day 42. The birds in each pen were weighed on days 0, 14, 28, 35, and 42. Feed was weighed before its addition to the feeder in each pen and remaining feed was weighed on feed transition days so that total feed intake could be calculated. Body weight, cumulative feed conversion ratio (cFCR), and cumulative feed intake (cFI) were determined at 0, 14, 28, 35, and 42 d. Feed conversion ratio was calculated by dividing the total feed intake per pen by the total body weight gain per pen, corrected for mortality. At the end of each experiment (42 d), following 8 h of feed withdrawal, 5 birds per pen were processed and deboned to determine carcass and breast yields.

#### 2.1.2. Experiment 3

Experiment 3 was approved by the Animal Ethic Committee of the Bangkok Animal Research Center (BARC) under protocol number AB21496A and was conducted following the guidelines for using animals for scientific purpose of the National Research Council of Thailand. Seven hundred and sixty-eight newly hatched Ross 308 male chicks were allocated to 2 treatments (CON and DUAL) with 24 replications per treatment, using 16 birds/pen in a randomized complete block design. A practical corn-soybean diet was formulated with three phases—starter (0–14 d), grower (14–35 d), and finisher (35 to 42 d). The diet was compliant with the nutrient requirements of the commercial strain recommendation (coccidiostat included in the diet formulation) and was used as the basal diet in all treatment groups, while the SCFP and EOC combination feed ingredient was added on top of the basal diet for the DUAL treatment group. The experiment was conducted in a closed house with a tunnel ventilation and evaporative cooling system and with solid-concrete-floor pens using rice hull as bedding material. Each pen measured 1.0 m × 1.5 m and was equipped with a tubular feeder and 3 nipple water drinkers. Feed and water were provided for *ad libitum* consumption. Birds were maintained under lighting and management programs according to commercial broiler breeder recommendations. All birds were vaccinated for Newcastle and Infectious Bronchitis diseases at 7 days and Gumboro disease at 14 days of age. At 37 days of age, feed and water were withdrawn from all pens for 12 h from 03.00 am until 3.00 p.m. and heat stress was induced from 9.00 a.m. to 3.00 p.m. by reducing pen space. Total pen feed consumption and body weight were measured weekly at 7, 14, 28, 35, and 42 days of age to determine body weight, cFCR, and cFI. Dead and culled birds were monitored and recorded daily. At the end of the trial (42 days of age), five birds from each pen with body weight close to the pen mean were slaughtered for breast yield measurement as well as carcass quality assessments such as footpad dermatitis, hock burn, and breast blister scored by trained panelist. Footpad dermatitis was measured by scores ranging from 0 to 4 (0 = smooth, no lesion; 1 = very small and superficial lesion, slight discoloration over a small surface; 2 = superficial lesion and discoloration of the footpad; 3 = moderate lesion, ulcers, and blood crust; 4 = severe lesions, ulcers, blood crust, hematomas, and deep wound). The scores of hock burn ranged from 0 to 2 (0 = Slight discoloration and/or small defects, but the absence of any lesion; 1 = very small and superficial lesion and slight discoloration over a small surface; 2 = superficial lesion and discoloration of the hock burn). The breast blister scores ranged from 0 to 1 (0 = absence and 1 = presence of breast blister).

#### 2.1.3. Statistical Analysis

For individual experiments, pens were used as experimental units and the data were analyzed using statistical software (SAS v9.4, Cary, NC, USA). Performance data from individual trials were analyzed using PROC MIXED with fixed effects of treatments and blocks as random effects. Livability and carcass quality data were analyzed using PROC GLIMMIX with the fixed effect of treatment and random effect of blocks. Averages across the data from the three experiments were analyzed using a PROC MIXED model with block nested in the study as a random effect and significant difference determined at *p* < 0.05. For livability and carcass quality data, PROC GLIMMIX was used with the fixed effect of treatment and block nested in the study as a random effect. The data are presented as least square means and standard error of the means (SEM) with significant statistical differences at *p* < 0.05.

## 3. Results and Discussion

### 3.1. Growth Performance

In Experiment 1, DUAL improved (*p* < 0.05) body weight, cFCR, and cFI, except at day 14 (Table 1). In Experiment 2, body weight gains appeared from day 28, with cFCR improvement (*p* < 0.05) at day 42. Early cFI was higher, but no difference (*p* > 0.05) was observed at market age. In Experiment 3, body weight gains were evident from day 14, and cFCR differences became significant (*p* < 0.05) from day 35. DUAL-fed birds also showed higher cFI, with a significant increase at day 42 (Table 1). Pooled analysis confirmed that DUAL improved (*p* < 0.05) body weight and cFCR at 14, 28, 35, and 42 days (Table 2). At market age, DUAL-fed birds had 5.5% higher body weight and a 5-point cFCR improvement compared to controls. cFI was also higher (*p* < 0.05) at 28, 35, and 42 days. The consistent improvements in body weight and feed efficiency highlight the efficacy of DUAL. The magnitude of differences, particularly the 5.5% increase in body weight and 5-point cFCR improvement at market age, demonstrates a clear advantage over controls. These findings align with earlier reports on SCFP improving broiler performance [18,19,20,36,37,38,39], turkeys [40,41], and layers/breeders [38,42,43]. They also support previous evidence that SCFP and EOC together enhance body weight and cFCR at 35 d [34]. No differences (*p* > 0.05) in livability were observed in individual trials (Exp 1—CON 96.76%, DUAL 95.80%; Exp 2—CON 92.51%, DUAL 91.70%; Exp 3—CON 96.23%, DUAL 96.74%) or in pooled analysis (CON 94.92%; DUAL 94.33%). The lack of differences in livability suggests that performance gains were not offset by health risks. Variations among experiments may reflect differing challenge conditions (such as re-used litter, heat stress, diets, etc.), yet the overall pattern shows robustness of DUAL under commercially relevant settings.

### 3.2. Mechanistic Insights on Gut Health and Performance Benefits

The complementary actions of SCFP and EOC likely underpin the observed performance improvements. SCFP supports distal gut microbiota, promoting *Lactobacillus* [24,44,45,46]. EOC acts in the upper gut [47], enhancing enzyme activity [30,32] and suppressing pathogens such as *E. coli* and *Salmonella* [31,33]. Their complementary distribution suggests synergy consistent with reports of SCFP reducing avian pathogenic *E. coli* lesion scores and pathogen loads [48] in broilers and improving turkey performance [41]. Preliminary DUAL studies also showed increased SCFA-producing bacteria at 21 d [49], which are linked to gut maturity and feed efficiency [50,51]. Collectively, these mechanisms suggest that DUAL accelerates microbiome development, optimizes gut health as well as nutrient utilization, and enhances growth performance.

### 3.3. Breast Meat Yield and Carcass Quality Improvements

**Figure 1:**
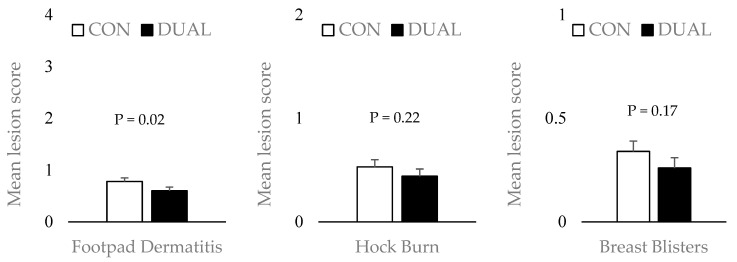
Carcass quality scores (footpad dermatitis, hock burn, and breast blisters) evaluated from Experiment 3 (*n* = 120 birds/treatment).

**Figure 2:**
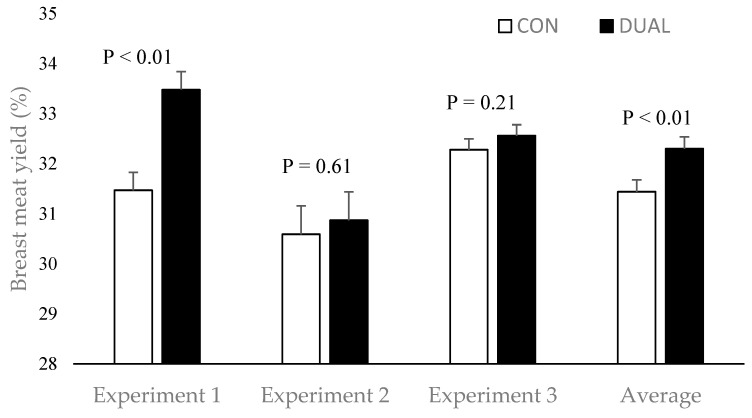
Breast meat yield from individual studies and pooled analysis across three different experiments (*n* = 120 birds/treatment/experiment).

In Experiment 3, DUAL-fed birds had lower (*p* < 0.05) footpad lesion scores and numerically better hock burn and breast blister scores (Figure 1). Combined processing data across all three experiments showed no difference (*p* > 0.05) in overall carcass yield between treatments (CON: 77.13% vs. DUAL: 76.80%). However, pooled breast meat yield was higher (0.86%, *p* < 0.05) in DUAL-fed birds (Figure 2). Carcass quality improvements, particularly reduced footpad lesions, may be linked to better gut health, lower excreta moisture. SCFP enhances gut integrity [19,37,52], while EOC supports nutrient absorption [30,32]. Together, they may reduce litter moisture, lowering skin lesion incidence. The increase in breast meat yield, though modest, suggests improved nutrient absorption and partitioning toward muscle deposition, possibly mediated by microbiome modulation. The exact mechanism of action of the combination in enhancing breast meat yield needs to be further investigated.

## 4. Conclusions

DUAL, a combination of SCFP and EOC, improved broiler performance across all trials. Birds fed DUAL showed higher body weight, better feed efficiency, and greater feed intake. Breast meat yield was increased, supporting growth and production. In Experiment 3, DUAL reduced footpad lesions, indicating carcass quality and welfare benefits. These effects are hypothesized to be related to faster gut microbiome maturation and improved nutrient use. Overall, DUAL is a promising nutritional strategy to enhance broiler health, productivity, and a potential reduction in footpad dermatitis. Further research is needed to clarify its mode of action.

## Figures and Tables

**Table 1:** Broiler performance from three independent experiments *.

		Experiment 1				Experiment 2				Experiment 3			
Age		CON	DUAL	SEM	*p*	CON	DUAL	SEM	*p*	CON	DUAL	SEM	*p*
14 d	BW	281.27	366.94	7.23	<0.01	534.89	530.22	5.56	0.37	528.04	536.10	3.40	<0.01
	cFCR	2.09	1.56	0.08	<0.01	1.08	1.08	0.01	0.72	1.09	1.08	0.01	0.24
	cFI	488.62	481.68	14.77	0.59	401.48	429.23	6.13	<0.01	527.52	533.50	2.42	0.09
28 d	BW	977.24	1193.13	27.91	<0.01	1771.95	1728.62	18.70	0.03	2026.05	2058.99	11.02	<0.01
	cFCR	1.88	1.67	0.04	<0.01	1.42	1.43	0.01	0.22	1.24	1.24	0.01	0.11
	cFI	1714.27	1866.84	20.17	<0.01	1854.65	1968.46	20.15	<0.01	2466.34	2495.19	14.29	0.01
35 d	BW	1495.76	1713.64	33.76	<0.01	2123.62	2184.98	20.25	0.04	2970.72	3031.37	15.52	<0.01
	cFCR	1.71	1.63	0.02	<0.01	1.65	1.61	0.01	0.06	1.33	1.31	0.00	<0.01
	cFI	2577.12	2809.50	31.41	<0.01	2832.88	2972.07	26.76	<0.01	3891.61	3921.27	22.02	0.09
42 d	BW	2207.88	2419.02	32.84	<0.01	2640.67	2782.39	50.71	<0.01	3771.97	3894.55	19.92	<0.01
	cFCR	1.69	1.65	0.01	0.03	1.74	1.65	0.02	<0.01	1.47	1.44	0.00	<0.01
	cFI	3751.09	4045.62	39.87	<0.01	4166.92	4119.37	148.34	0.82	5461.76	5524.76	29.18	<0.01

* Experiment 1: *n* = 24 reps/treatment with 20 birds/reps (*n* = 480 birds/treatment); Experiment 2: 24 reps/treatment with 25 birds/treatment (*n* = 600 birds/treatment); Experiment 3: 24 reps/treatment with 16 birds/reps (*n* = 384 birds/treatment); in all experiments reps or pens were used as experimental units. CON—Control; DUAL—Biostrong™ Dual; SEM—Standard error of the means; *p*—*p* values; BW—Body weight; cFCR—Cumulative feed conversion ratio; cFI—Cumulative feed intake.

**Table 2:** Pooled analysis of body weight, cumulative feed conversion ratio (cFCR), and cumulative feed intake (cFI) across three different experiments at different time points.

Age in Days	Body Weight (g)				cFCR				cFI (g)			
	CON	DUAL	SEM	*p*	CON	DUAL	SEM	*p*	CON	DUAL	SEM	*p*
0	43.01	42.81	0.12	0.08	-	-	-	-	-	-	-	-
14	445.28	474.95	3.52	<0.01	1.4245	1.2463	0.03	<0.01	531.39	529.68	5.34	0.7403
28	1574.23	1642.73	11.31	<0.01	1.515	1.4513	0.01	<0.01	2195.4	2248	11.87	<0.01
35	2191.35	2303.15	14.79	<0.01	1.5611	1.5159	0.01	<0.01	3287.06	3368.69	15.46	<0.01
42	2852.19	3009.68	21.25	<0.01	1.6352	1.5816	0.01	<0.01	4465.61	4575.19	32.82	<0.01

## Data Availability

Data will be made available from the corresponding author upon reasonable request.
